# Transdermal delivery of FITC-Dextrans with different molecular weights using radiofrequency microporation

**DOI:** 10.1186/s40824-020-00201-7

**Published:** 2020-12-09

**Authors:** Guk Young Ahn, Hae-Seok Eo, Dongwon Kim, Sung-Wook Choi

**Affiliations:** 1 Department of Biomedical-Chemical Engineering, The Catholic University of Korea, 43 Jibong-ro Wonmi-gu, Bucheon-si, Gyeonggi-do 14662 Republic of Korea; 2 LG electronics, 19 Yangjae-daero 11-gil, Seocho-gu, Seoul, 06772 Republic of Korea

**Keywords:** Microporation device, Human cadaver skin, Transdermal drug delivery

## Abstract

**Background:**

Transdermal delivery is of great importance for the effective delivery of bioactive or therapeutic agents into a body. The microporation device based on radiofrequency can be used to enhance delivery efficiency by removing the epidermis layer.

**Methods:**

The micropores were developed on pig skin and human cadaver skin with dermal and epidermal layers by the microporation device. The regeneration of micropores in the human cadaver skin caused by microporation was confirmed using an optical microscope and haematoxylin/eosin (H&E) staining. The permeability of fluorescein isothiocyanate-dextrans (FITC-dextrans) with different molecular weights through the pig and human cadaver skins were measured using Franz diffusion cell.

**Results:**

The optical image and histological analysis confirmed that the micropores on the skin were recovered over time. The enhanced permeability through micropores was confirmed by Franz diffusion cell. The lower molecular weight of FITC-dextran permeated more on both human and pig skin. In addition, the permeation rate was higher in pig skin than in human skin.

**Conclusions:**

We believe that the microporation device can be used as a potential technique for effective transdermal drug delivery.

## Background

Transdermal drug delivery is an attractive alternative that addresses the limitations of oral and parenteral routes of drug administration; it enables controlled release and long-term systemic drug delivery through the skin, avoids hepatic first-pass effect, avoids gastrointestinal drug degradation, reduces discomfort and trauma resulting from hypodermic injections, and prevents safety hazards associated with dangerous medical waste from needles [1, 2]. However, the currently available transdermal systems are limited by low drug permeability across the skin due to the lipophilic barrier function of the stratum corneum (SC), the outermost skin layer of the skin [3–5]; the methods favor low molecular mass, and lipophilic molecules, making it difficult to exploit the transdermal route for large and water-soluble molecules such as proteins and peptides. To overcome these limitations, microporation devices that act as therapeutic vehicles for delivering drug molecules across the skin barriers have been developed. This method utilizes promising strategies that have made a significant impact in transdermal delivery [6], they include: iontophoresis, microneedles, sonophoresis, radiofrequency (RF), and chemical enhancers [7–11]. Radiofrequency is a well-known medical technique with expanded application in electrosurgery for laparoscopic medical procedures and in dermatology, for sebaceous glands ablation in acne treatment [12, 13], and treatment of photoaging skin symptoms, including wrinkles and hyperpigmentation [14]. To improve drug permeability, micropore ablation using RF is performed by passing an alternating electrical current through the skin at a frequency higher than 100 kHz. Micropores are produced by the arrangement of microelectrodes on the skin at precise dimensions, then RF energy generated by alternating currents induces ionic vibrations between electrodes with positive or negative charges. These vibrations heat the skin tissue, triggering water vapor and cell ablation, forming microchannels from the SC through to the outer dermis. Immediately after formation, the microchannels are filled with interstitial fluid, making them hydrophilic, and a suitable drug delivery system [15]. The effectiveness of the RF microporation technique in transdermal drug delivery of macromolecules and hydrophilic agents such as peptides, hormones, and vaccines has been demonstrated extensively in previous studies [16–18]. However, in vitro investigations are very few. In this study, we investigated the effectiveness of RF microporation technology in vitro, by evaluating the permeation of fluorescent-conjugated dextran (FITC-dextran) of various molecular weights through the human and pig skin after microporation, using a transdermal diffusion cell system. In addition, we evaluated the regeneration of SC after microporation. Because of the recent animal protection rules which have restricted animal experiments [19], we used human cadaver skin as an alternative to animals. Human cadaver skin has been confirmed as alive skin tissue through analysis of its morphology and enzymes, and its usefulness as an alternative skin membrane in drug permeation experiments has been verified [20].

## Materials and methods

### Materials

FITC-dextran molecular weights; 4, 10, and 20 kDa molecular weight were purchased from Sigma (St. Louis, MO, USA). Dulbecco’s Phosphate-Buffered Saline (DPBS) was purchased from Welgene (Gyeongsan, Korea). The skin culture medium was purchased from Biopredic International (Saint-Grégoire, France). 10% neutral buffered formalin was purchased from Hisko (Gunpo, Korea).

### Equipment

The skin poration was performed using the microporation device (LG electronics, Korea). The measurements of the transepidermal water loss (TEWL) were taken using the Tewameter (Courage-Khazaka Electronic GmbH, Cologne, Germany) to evaluate the skin barrier function after microporation. The Transdermal diffusion cell system (DHC-6TD, LOGAN instrument, USA) was used to assess FITC-dextran permeability in vitro.

### In vitro human cadaver skin regeneration after microporation

Human cadaver skins (fresh human full-thickness skin disc, Ø 12 ~ 20 mm) were supplied by Biopredic International (Saint-Grégoire, France). The skins were from the abdomen of a 39-year-old caucasian woman (BMI: 24). One day before the experiment, the skin was stabilized in the skin culture medium. Barrier integrity was examined using Tewameter to confirm the poration of human cadaver skin using a microporation device. TEWL measurements were performed before and 30 min after microporation. Microporation was performed 2, 5, and 10 times on each sample. Each microporated sample was incubated in the skin culture medium. Pore closure examination was performed using an optical microscope (BX43, Olympus, Japan) immediately (time 0), 4, 8, and 24 h following device application. Each skin pores was captured using a microscope and the obtained images were analyzed using ImageJ® software (National Institutes of Health, Bethesda, USA).

### Histological characterization of skin pore closure

After the pore closure examination, each sample was stored in a 10% neutral buffered formalin awaiting haematoxylin/eosin (H&E) staining. These studies were carried out at the Hisko (Gunpo, Korea). H&E stained images were analyzed using Image J^®^ software.

### In vitro transdermal delivery after microporation

The human and pig skin permeability of FITC-dextran (M.W. 4, 10, and 20 K) was measured using the Transdermal diffusion cell system. FITC-dextran solution was prepared at 1 mM in PBS. Human skins (Full-thickness human skin) were supplied by HansBiomed. Corp. (Seoul, Korea). The human skins were from the back or thigh of a 24-year-old woman. Pig skins (Micropig® Franz cell membrane) were supplied by Medi Kinetics Co., Ltd. (Pyeongtaek, Korea). Microporation was performed 2 times on each sample. TEWL measurements were taken 30 min after microporation of the human and pig skin. The human and pig skin samples were placed in the receiver chamber of the Transdermal diffusion cell system with the SC facing up and the donor chamber was fixed in place. The receptor chamber was filled with PBS. FITC-dextran solution (600 μL) was added to the donor chamber. The Transdermal diffusion cell system was protected from light. The receptor phase was collected immediately (time 0) and 2, 4, 8, 12, and 24 h. This receptor phase solution was transferred to a 96-well plate and the skin-permeated FITC-dextran was measured using a microplate reader (Molecular Devices, Co. Ltd., Sunnyvale, CA, USA) at 405 nm.

### Statistics

Quantitative data are presented as the mean ± standard deviation, and comparisons were carried out using one-way ANOVA (Systat Software Inc., Chicago, IL, USA). Differences were considered statistically significant at *p* < 0.05.

## Results

**Fig. 1:**
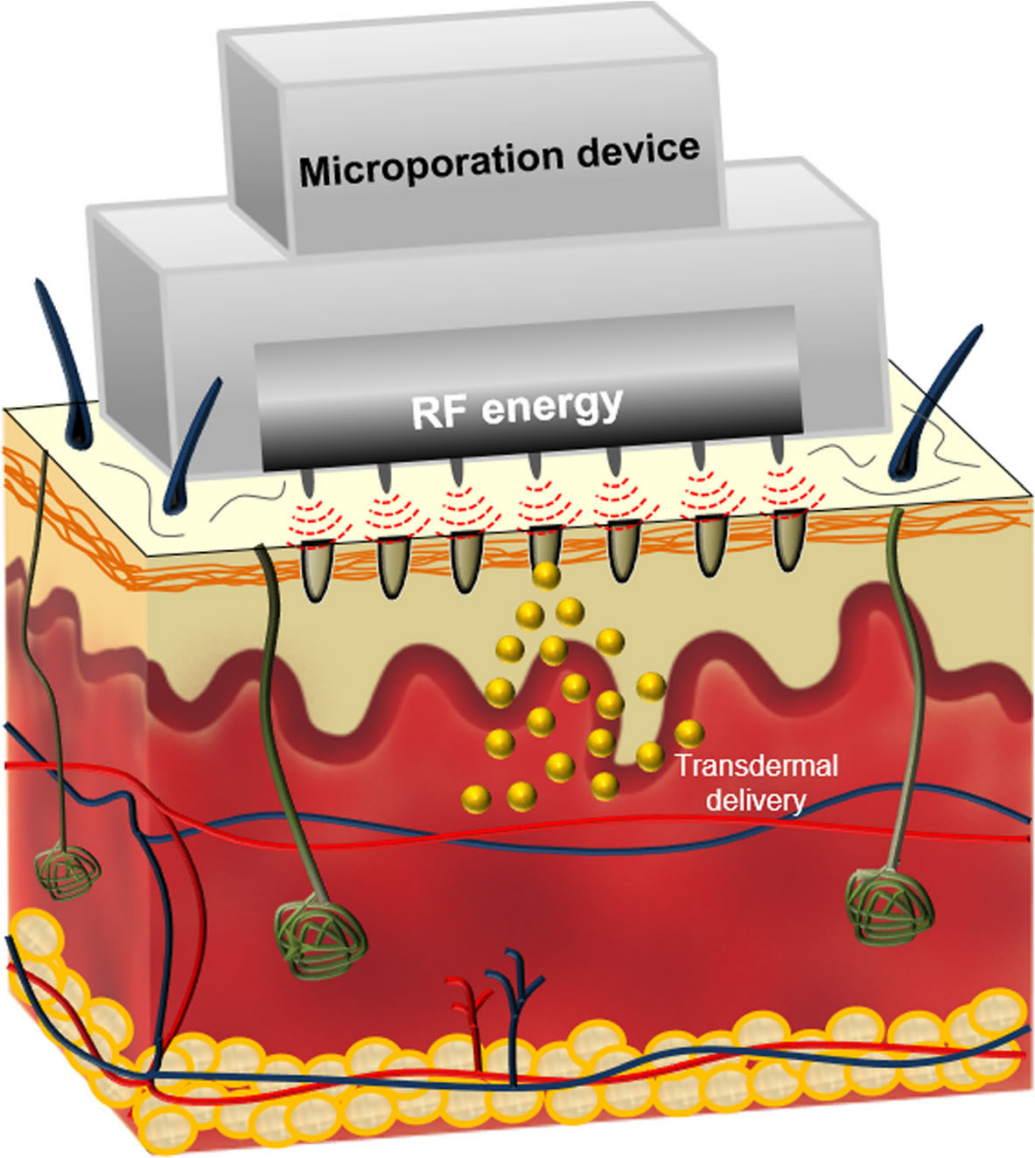
Schematic illustration of transdermal delivery after the application of the microporation device

Figure 1 shows a schematic illustration of transdermal delivery after the application of the microporation device. The skin was treated with the RF-based microporation device, inducing micropore ablation through alternating electrical current. This device can generate microchannels from the SC through to the outer dermis and allow transdermal delivery of active agents.

**Fig. 2:**
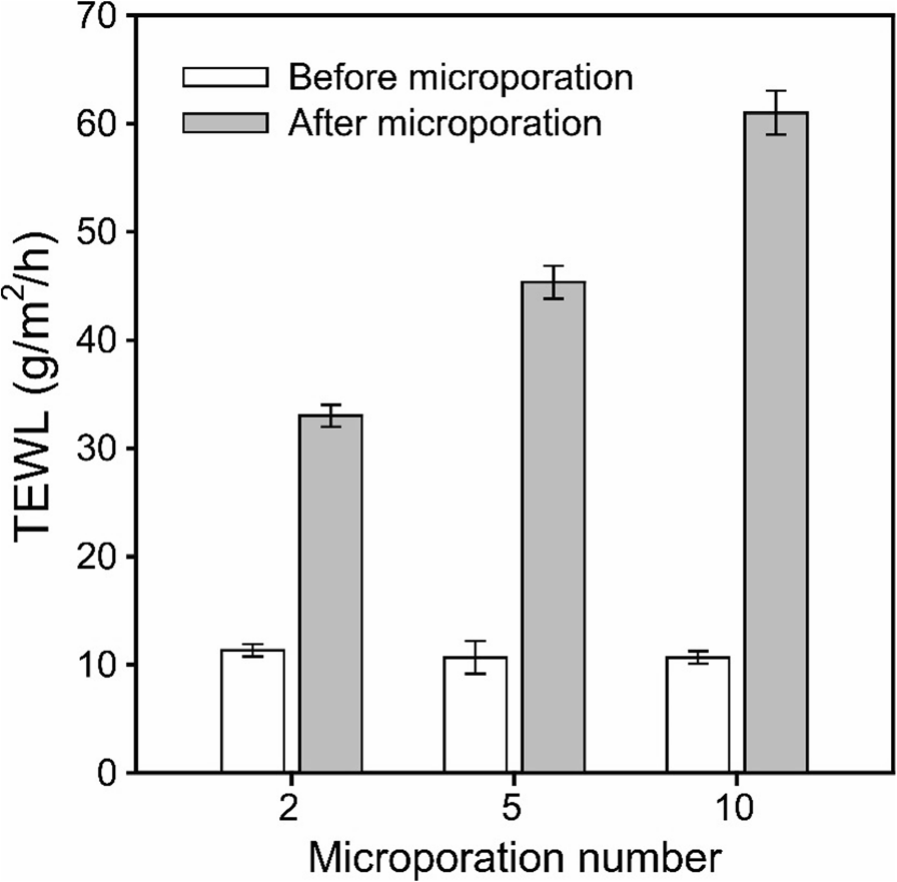
TEWL values before and immediately after microporation on human cadaver skin according to the number of times microproration was done (*n* = 3)

Figure 2 shows TEWL values measured before and immediately following the treatment of human cadaver skin with the microporation device. To confirm the extent of skin damage and regeneration after microporation, porations were performed 2, 5, and 10 times. TEWL measurements were taken to assess the integrity of the skin barrier and the extent of SC disruption. After microporation, the TEWL values of the human cadaver skin increased from 11.3 ± 0.6 to 33.0 ± 1.0, 10.7 ± 1.5 to 45.3 ± 1.5, and 10.7 ± 0.6 to 61.0 ± 2.0 g/m^2^/h when the microporation was performed 2, 5, and 10 times, respectively.

**Fig. 3:**
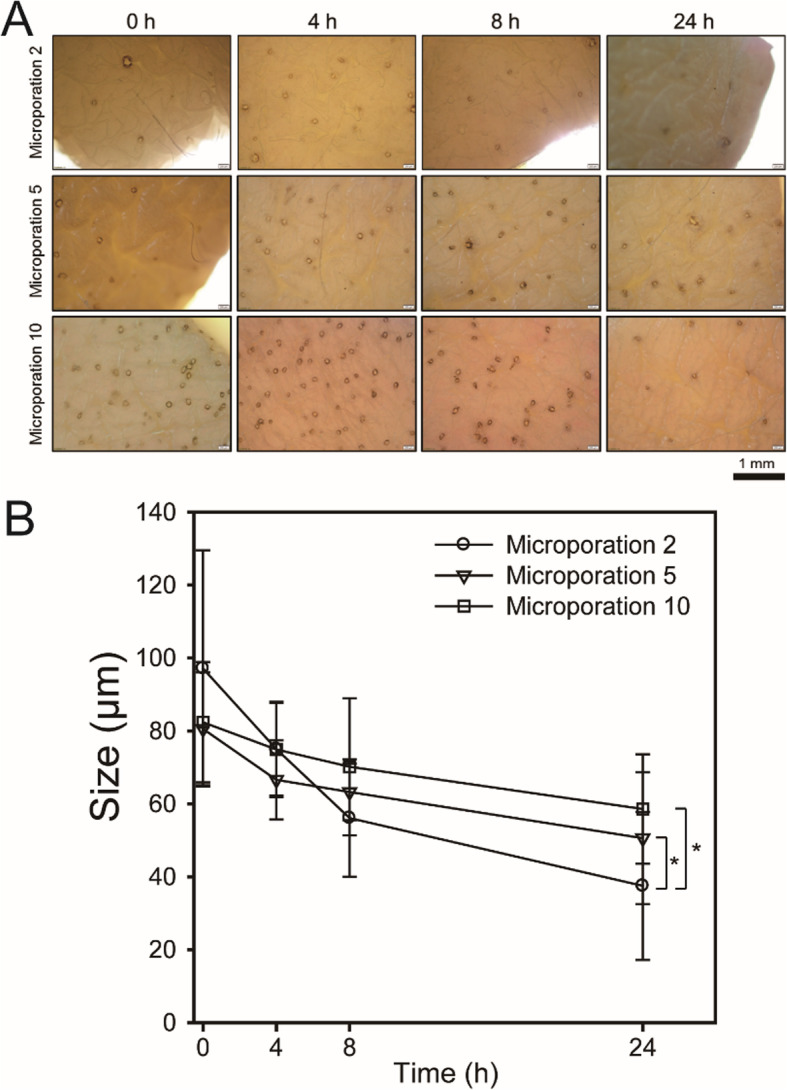
**a** Microscopy images of micropore and **b** quantified data using ImageJ after 0 (immediately), 4, 8, and 24 h according to the number of times microproration was done (*n* = 150, *: *p* < 0.05)

Figure 3 shows a magnified image of human cadaver skin with microchannels formed by the microporation device (250 kHz) and the skin closure over time. Immediately after 2, 5, and 10 times microporations, 97.14 ± 32.39, 80.57 ± 15.51, and 82.39 ± 16.49 μm sizes of micropores were formed on the skin, respectively. 4 h after microporation, the sizes of the micropore decreased slightly. After 8 h, the micropore became more distorted from the previous circle, suggesting that the micropores were closing. After 24 h the micropores were either closed or reduced further to smaller sizes, 37.50 ± 20.41, 50.57 ± 18.07, and 58.58 ± 14.99 μm, for 2, 5, and 10 times microporation, respectively. These results indicate that healing and regeneration occurred in the human cadaver skin after the microporation.

**Fig. 4:**
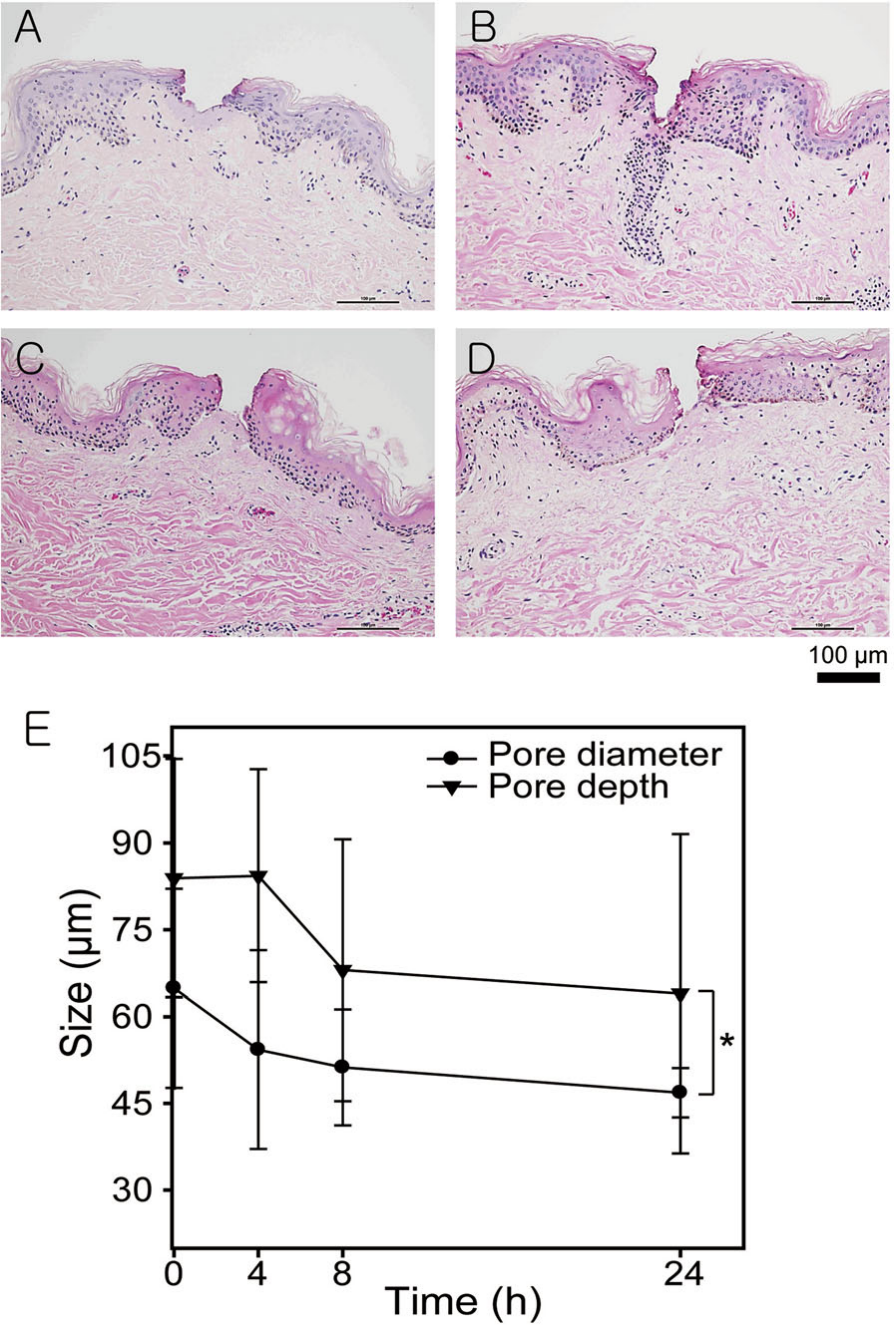
**a**-**d** Haematoxylin/eosin-stained histological sections of human cadaver skin sample and **e** quantified data using ImageJ for different time periods after microporation. **a** Immediately after microporation, **b** 4 h, **c** 8 h, and **d** 24 h (*n* = 5, *: *p* < 0.05)

Figure 4 shows the histological analysis of micropores in human cadaver skin. The micropore formed at the SC through to the epidermis, enabling drug delivery. 24 h after microporation, the pore sizes reduced from 64.88 ± 17.20 μm (0 h) to 48.85 ± 4.27 μm (24 h), and the pore depths reduced from 83.91 ± 20.56 μm (0 h) to 63.95 ± 27.61 μm (24 h). These results suggest that fibroblasts proliferate and migrate under the SC, and regenerate the skin.

**Fig. 5:**
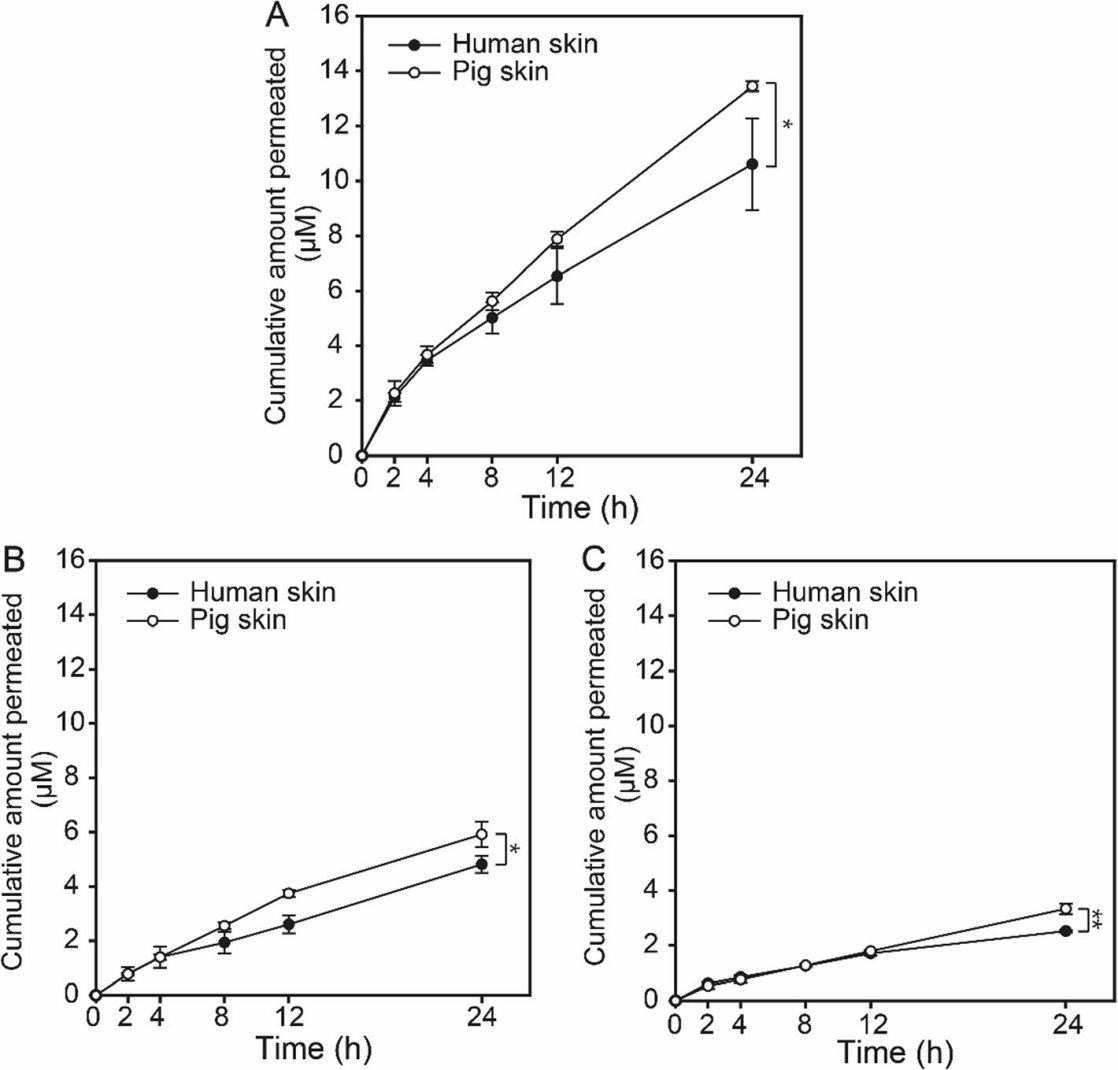
Cumulative amount of FITC-dextran permeated after microporation on the human and pig skin for FITC-dextran M.W.. **a** M.W. 4 K, **b** M.W. 10 K, and **c** M.W. 20 K. * shows a significant difference between the M.W. 4 K and M.W. 20 K groups (*n* = 3, *: *p* < 0.05, **: *p* < 0.01)

Table 1 shows TEWL values measured immediately after the microporation of human and pig skins. Increased TEWL values are evidence of micropores formation. These results are in agreement with a previous study which suggested that the microporation using radiofrequency causes disruption of the SC, enabling the percutaneous permeation of the FITC-dextran [21]. Figure 5 shows the cumulative amount of the FITC-dextran (M.W. 4 K, 10 K, and 20 K) permeated through the human, and pig skin after microporation. After 24 h, the cumulative permeation of FITC-dextran M.W. 4 K was 10.61 ± 1.68 and 13.44 ± 0.18 μM in human and pig skin, respectively. The cumulative permeation of FITC-dextran 10 K was 4.82 ± 0.32 μM, in the human skin and 5.92 ± 0.46 μM in the pig skin. With FITC-dextran 20 K, cumulative permeation in the human skin was 2.53 ± 0.48 μM, and 3.34 ± 0.19 μM in the pig skin, which significantly lower than FITC-dextran 4 K.

**Table 1:**
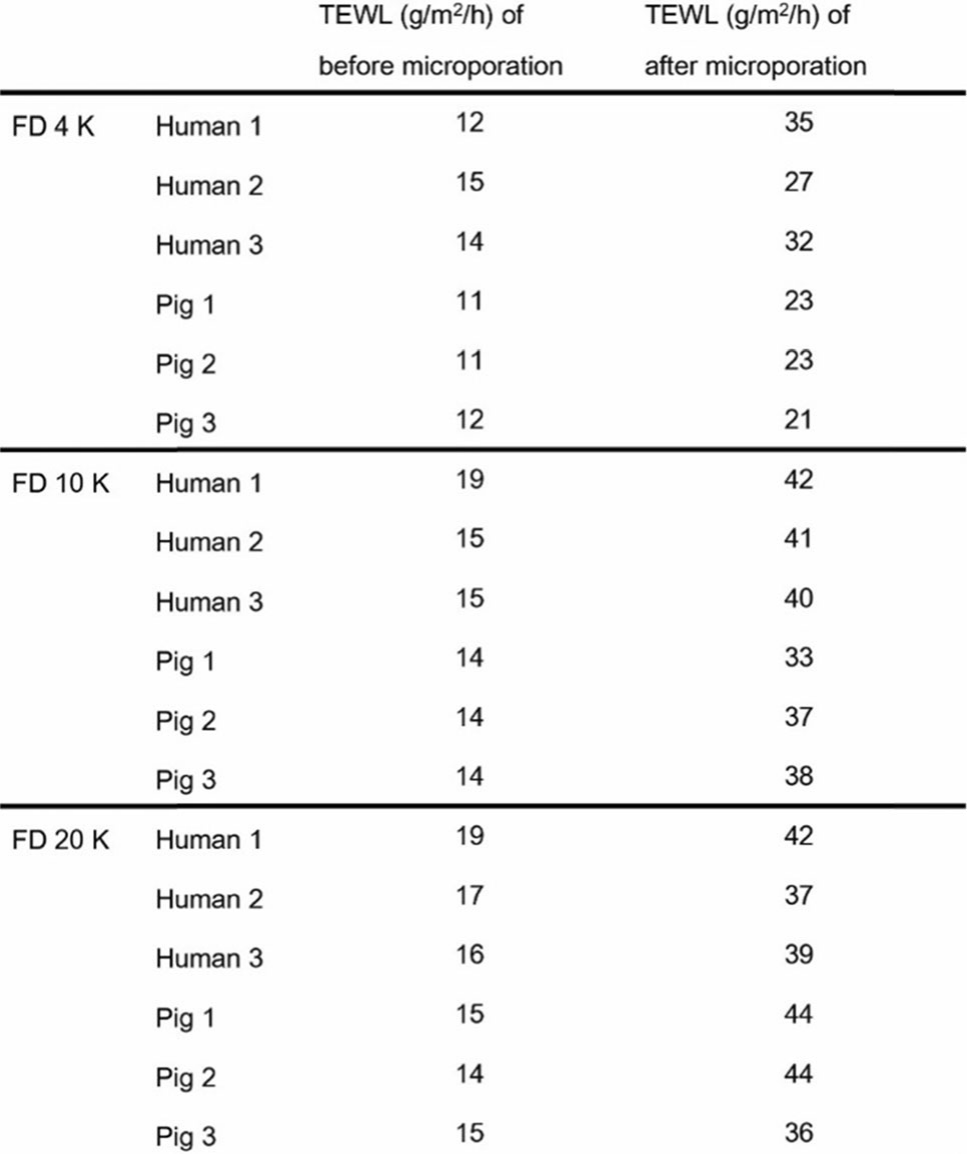
TEWL values before and immediately after microporation of the human and pig skin

## Discussion

In this work, the human cadaver skin was treated to confirm the skin regeneration after the application of the microporation device. Microporations were performed 2, 5, and 10 times and the skin damage was confirmed using the TEWL measurement. Normally, the skin loses very little water loss whereas damaged skin loses more [22]. The increased TEWL values which were proportional to the number of microspores indicate SC disruption and formation of microchannels in the skin.

The micropores of the human cadaver skin were regenerated according to the time. Keratinocytes adjacent to the wound site induced by the microporation in the epidermis undergo a series of modifications that allow their proliferation and migration to the wound. These modifications include degradation of hemidesmosomes attached to the dermis, degradation of desmosomes linked to adjacent cells, contraction of intracellular tonofilament, and lamellipodia formation. The regeneration process of the skin is regulated by growth factors such as keratinocyte growth factor and fibroblast growth factor, which trigger the proliferation and migration of cells [23, 24]. However, the results did not show significant regeneration of micropore compared to previous in vivo studies which reported significant regeneration [25]. These results could be attributed to the differences in the in vivo and in vitro skin nutrients supply; with in vivo studies, the skin tissue is supplied nutrients directly from the blood vessels, but the human cadaver skin is supplied from the media, therefore, the skin tissue regeneration efficiency is low [26–28]. However, considering the trauma and unethical practices associated with animal experiments, in vitro studies with human cadaver skin offers an ethical and viable alternative. Although we didn’t conduct assay for inflammation, we could not find any inflammation symptoms such as redness, blisters, cracked, and thickening.

The dextran has the advantage in terms of low cytotoxicity, excellent biocompatibility, and water-solubility. Therefore, it is suitable as model molecules with different molecular weights for transdermal drug delivery [29]. In our present study, microporated skin permeated insufficient amount of 20 K FITC-dextran, suggesting that enhancement of permeability was unsuccessful with macromolecules of more than 20 K M.W.. In addition, microporated pig skin permeated larger amounts of FITC-dextran than human skin, this can be attributed to the differences in structure between the human and pig skin; the pig skin layer is thinner (SC, 8–13 μm) than the human skin (SC, 10–17 μm) and the pig hair follicle is longer (38–71 μm) than the human hair follicle (18 μm). These structural differences can significantly affect FITC-dextran delivery due to even after microporation [30]. Based on these results a difference of approximately 10–15% between the amounts of drugs permeated through the human skin and pig skin models should be considered during human clinical trials.

## Conclusions

In summary, we demonstrated effective enhancement of skin permeability and delivery of macromolecules lower than 20 K M.W., and regeneration of human cadaver skin after microporation with an RF-based microporation device. In vitro studies with human cadaver skin is a viable, ethical, and economical alternative for in vivo animal studies. In the future, we plan to investigate the possibility of enhancing the delivery of other macromolecular drugs such as peptides and siRNAs using the RF-based microporation device. We will also conduct further studies on the regeneration of human cadaver skin and evaluate the possibilities of clinical trials.

## Data Availability

For data requests, please contact the authors.
